# Complement factor H binding of monomeric C-reactive protein downregulates proinflammatory activity and is impaired with at risk polymorphic CFH variants

**DOI:** 10.1038/srep22889

**Published:** 2016-03-10

**Authors:** Blanca Molins, Pablo Fuentes-Prior, Alfredo Adán, Rosa Antón, Juan I. Arostegui, Jordi Yagüe, Andrew D. Dick

**Affiliations:** 1 Institut d’Investigacions Biomèdiques Agustí Pi i Sunyer (IDIBAPS), Hospital Clínic de Barcelona, 08028 Barcelona, Spain; 2 Molecular Bases of Disease, Biomedical Research Institute Sant Pau (IIB Sant Pau), 08025 Barcelona, Spain; 3 Universitat Autònoma de Barcelona, 08193 Bellaterra (Cerdanyola del Vallès), Spain; 4 Department of Immunology-CDB, Hospital Clínic-IDIBAPS, 08028 Barcelona, Spain; 5 Academic Unit of Ophthalmology, School of Clinical Sciences and School of Cellular and Molecular Medicine, University of Bristol, Bristol, BS8 1TH, UK; 6 National Institute for Health Research (NIHR) Biomedical Research Centre at Moorfields Eye Hospital and University College London Institute of Ophthalmology, London, EC1V 2PD, UK

## Abstract

Inflammation and immune-mediated processes are pivotal to the pathogenic progression of age-related macular degeneration (AMD). Although plasma levels of C-reactive protein (CRP) have been shown to be associated with an increased risk for AMD, the pathophysiological importance of the prototypical acute-phase reactant in the etiology of the disease is unknown, and data regarding the exact role of CRP in ocular inflammation are limited. In this study, we provide mechanistic insight into how CRP contributes to the development of AMD. In particular, we show that monomeric CRP (mCRP) but not the pentameric form (pCRP) upregulates IL-8 and CCL2 levels in retinal pigment epithelial cells. Further, we show that complement factor H (FH) binds mCRP to dampen its proinflammatory activity. FH from AMD patients carrying the “risk” His402 polymorphism displays impaired binding to mCRP, and therefore proinflammatory effects of mCRP remain unrestrained.

Age-related macular degeneration (AMD) is the primary cause of irreversible vision loss among the ageing population worldwide. The disease affects up to 1.75 million individuals alone in the United States, and this number could increase up to 3 million by 2020^1^,^2^,^3^. Worldwide, the projected number of people with AMD in 2020 is 196 million (95% CrI 140–261), which increases to 288 million in 2040 (205–399)^4^. Local inflammation and immune-mediated processes play a central role in AMD pathogenesis^5^. Proteomic and histochemical analysis of ocular drusen, the hallmark deposits of AMD, have shown that these deposits contain inflammatory proteins and complement components that mediate local inflammation^6^,^7^. Furthermore, polymorphisms in a gene essential for the regulation of complement activation, *CFH*, are an important risk factor for developing AMD^8^,^9^. These and other findings have stimulated research to understand the role of immune responses in the development of the disease.

C-reactive protein (CRP) is the prototypical acute-phase reactant and an active regulator of the innate immune system. It is considered to be a serum biomarker for chronic inflammation, heart disease and, more recently, AMD^10^,^11^,^12^. CRP has been identified in ocular drusen and other subretinal pigmented epithelium deposits^13^,^14^, as well as in the choroid, but little is known about its function in the context of AMD. Among the multiple functions ascribed to CRP are activation of the classical complement pathway and inactivation of the alternative pathway^15^. In addition, in the context of atherosclerosis, CRP upregulates the expression of adhesion molecules, and increases cytokine release from endothelial cells, neutrophils and macrophages^16^,^17^,^18^.

In plasma, CRP exists as a cyclic non-covalent pentamer of 125-kDa composed of five identical Ca^2+^-stabilized subunits, which are placed in the corners of a regular pentagon^19^. (See Supplemental Figure 1A for a representation of the three-dimensional (3D) crystal structure of pentameric CRP (pCRP); notice that a phosphocholine molecule is bound to each of the five independent binding sites)^20^. However, oxidative stress, low pH, and bioactive lipids from activated or damaged cells can dissociate pCRP into its 23-kDa subunits^21^,^22^,^23^,^24^. This poorly soluble, tissue-based monomeric form, mCRP, possesses distinct biological functions compared to pCRP^23^. For instance, mCRP induces proinflammatory responses in neutrophils and endothelial cells, in contrast to the anti-inflammatory functions of pCRP^25^,^26^,^27^. Further, data shows that, unlike pCRP, mCRP elicits a prothrombotic and proinflammatory response in platelets and monocytes^28^,^29^,^30^.

Complement factor H (FH) is a major inhibitor of the alternate complement pathway. FH regulates complement activation in plasma, host cells and tissue, in particular at sites of tissue inflammation, following injury or during degeneration^31^. The protein is essentially comprised of 20 tandem Sushi domains, also known as short consensus repeat (SCR) or complement control protein (CCP) modules. A common polymorphism in the *CFH* gene (c.1277T>C, p.Tyr402His) is highly associated with the development of AMD; the CFH p.Tyr402His variant (in following termed CFH_H402_) increases the risk for AMD ∼2–4-fold for heterozygous and 5–7-fold for homozygous individuals^32^,^33^,^34^. The exchanged residue is located in FH domain SCR7, which mediates cell surface binding through interactions with heparan sulfate chains^35^,^36^. FH is in addition known to bind CRP, but there is an ongoing controversy regarding the relevance of the monomeric and pentameric forms in this regard. For instance, two separate binding sites for pCRP were located on domains SCR4–6 and SCR16–20, respectively^36^. On the other hand, FH showed strong binding to denatured, monomeric CRP, rather than to the native multimeric form^37^,^38^. Of particular note, the non-risk associated variant FH_Y402_ binds CRP more strongly than the H402 risk variant^39^,^40^,^41^. As a consequence, individuals who are homozygous for the latter show 2.5-fold higher CRP levels in the retinal pigment epithelium (RPE)–choroid layer compared with those homozygous for the non-risk variant^40^.

Although mCRP has been localized within retinal tissues, the contribution to immune responses and to AMD onset and progression has not been clarified. The data supports our hypothesis that mCRP contributes to the development of AMD through direct proinflammatory effects on retinal pigment epithelial cells, and that this proinflammatory activity is unchecked in subjects with the “at risk” Tyr402His polymorphism in CFH, due to an impaired interaction with mCRP.

## Results

### Monomeric but not pentameric CRP induces *IL8* and *CCL2* gene expression and protein secretion

**Figure 1:**
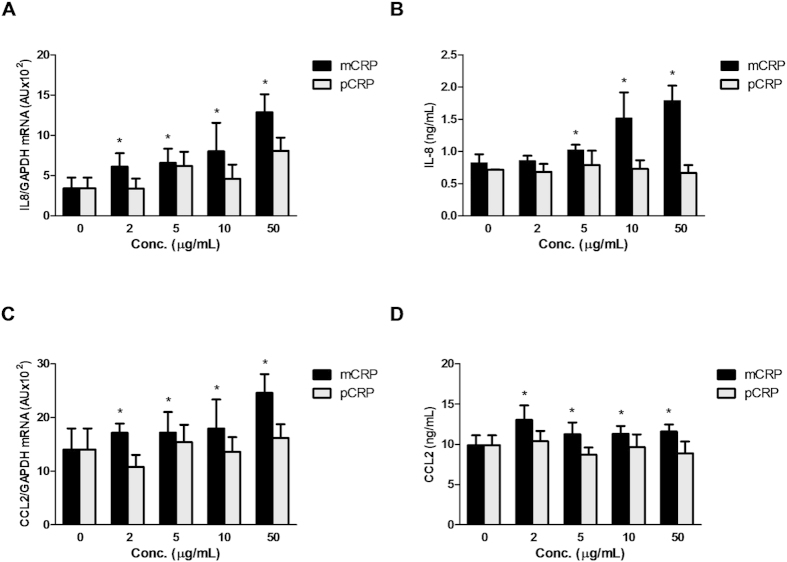
Monomeric, but not pentameric CRP isoforms stimulate IL-8 and CCL2 expression. ARPE-19 cells were stimulated with the indicated concentrations of CRP isoforms for 24 h. Gene expression levels of *IL*8 (**A**) and *CCL2* (**C**) as well as secreted concentrations of these cytokines (**B**,**D**) were determined by real-time PCR and ELISA, respectively (n = 7). Statistical analysis was performed by ANOVA (*P < 0.05).

To determine the effect of CRP isoforms on the expression of inflammatory mediators, ARPE-19 cells were exposed for 24 h to different concentrations of either mCRP or pCRP. mRNA levels of *IL8* and *CCL2* were determined by quantitative RT-PCR, and protein concentrations of both cytokines in the supernatants were quantified by ELISA. As observed in Fig. 1A, mCRP significantly and dose-dependently increased *IL8* mRNA expression (*P* < 0.05), reaching statistical significance at a concentration of 2 μg/mL. By contrast, pCRP was unable to induce IL-8 expression at any concentration tested. Consistent with the stimulation of *IL8* gene expression, mCRP significantly and dose-dependently augmented the secretion of IL-8 (Fig. 1B). By contrast, pCRP was unable to induce IL-8 secretion at any concentration tested. Similar to the effect on IL-8, only mCRP significantly increased *CCL2* mRNA expression levels in a dose-dependent manner, reaching significance at 5 μg/mL (Fig. 1C). Concentrations of the secreted cytokine were also significantly higher in cultures treated with the monomeric CRP form, as compared to pCRP (Fig. 1D). To rule out the possibility that pCRP at equal molar concentrations as mCRP (and thus at a five-fold higher concentration of binding sites) could exert a similar response as the monomeric form, we determined mRNA and secreted protein levels of IL-8 from ARPE-19 cells in response to increasing concentrations of m/pCRP (from 0 to 2.5 mM). As shown in Supplementary Figure S2, the monomeric form significantly increased *IL8* gene expression and protein secretion at concentrations above 0.25 mM while pCRP was unable to stimulate IL-8 secretion at any concentration tested.

Fcγ receptors have been postulated to play a role downstream of CRP binding^25^,^26^. We addressed this issue by blocking CD16 (Fcγ-RIII) or CD32 (Fcγ-RIIa) receptors with specific mAbs. Blockade of CD32 did not prevent mCRP-induced upregulation of IL-8 and CCL2 in ARPE-19 cells (Supplementary Figure S3). By contrast, blockade of CD16 prevented CCL2, but not IL-8 upregulation. To further investigate whether MAPK pathways were involved in mCRP-induced upregulation of cytokine expression, ARPE-19 cells were treated with p38 inhibitor SB-203580, MEK inhibitor U0126 or JNK inhibitor SP-600125 for 30 min prior to mCRP treatment. mCRP-induced secretion levels of IL-8 and CCL2 were partially reduced by SB203580 and completely reverted by the JNK inhibitor. However, the MEK inhibitor did not significantly influence secretion of any of the cytokines (Supplementary Figure S4).

### Conditioned medium from mCRP-treated ARPE-19 cells stimulates PBMC migration

**Figure 2:**
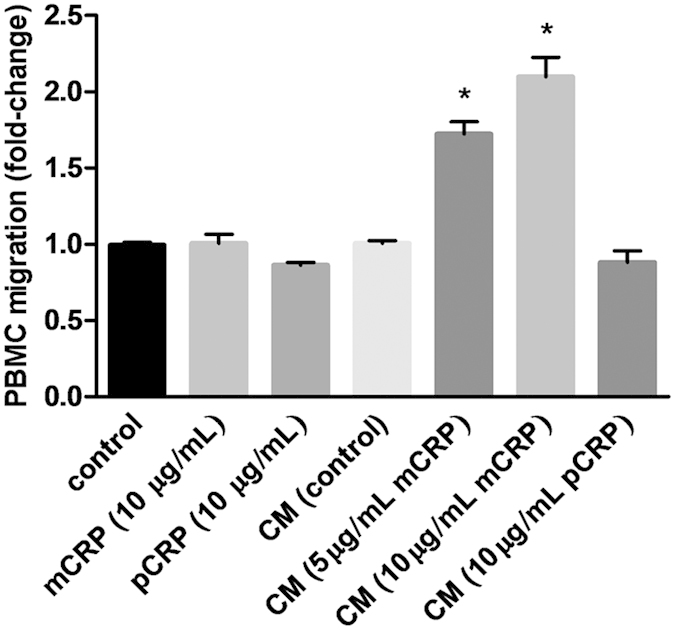
Cytokines secreted by mCRP-treated ARPE-19 cells potently stimulate PBMC migration. Chemotaxis assay was performed using Transwell chambers. PBMC from healthy donors were seeded into the upper wells, while the lower chambers contained either RPMI medium supplemented with 10 μg/mL mCRP or pCRP, or conditioned medium from ARPE-19 cells stimulated with the indicated concentrations of CRP isoforms, and diluted 1:1 with RPMI. The results of these experiments are expressed as fold-change vs. control ± SEM after 3 h incubation (n = 4). Statistical analysis was performed by ANOVA (*P < 0.05).

Since mCRP induced secretion of proinflammatory and chemotactic mediators from ARPE-19 cells, we wished to evaluate the functional consequences of this upregulation. For this purpose, we performed assays to evaluate whether the conditioned medium of CRP-treated ARPE-19 cells had a chemotactic effect on healthy PBMC. We observed that none of the CRP isoforms *per se* had chemotactic activity on PBMC, as the number of migrated cells did not change when mCRP or pCRP were added to RPMI1640 medium (Fig. 2). Conditioned medium from non-stimulated ARPE-19 cells did not show chemotactic properties, as the number of cells migrated after 3 h stimulation was similar to the effect produced by RPMI1640 medium alone. On the contrary, conditioned medium from mCRP-stimulated but not from pCRP-stimulated cells significantly increased PBMC migration (*P* < 0.05). These observations suggest that mCRP confers a proinflammatory phenotype to ARPE-19 cells, which further induces PBMC migration.

### Proinflammatory cytokines do not induce CRP expression in ARPE-19 cells

To elucidate whether CRP is expressed locally by RPE cells, ARPE-19 cells were cultured in the presence of different proinflammatory cytokines for 24 to 48 h, and *CRP* mRNA was analyzed by RT-PCR. No evidence of *CRP* gene transcription was detected in ARPE-19 cells after these incubation times. When transcriptional data was analyzed using low-stringency PCR baseline levels, small quantities of *CRP* mRNA were detected in ARPE–19 cells, especially upon stimulation with IL-17 (Supplementary Figure S5). However, these levels are almost indistinguishable from nonspecific background amplification. Similarly, CRP levels in the supernatants of ARPE-19 treated cells were below the detection limit (0.4 ng/mL) under all tested conditions.

### FH prevents the proinflammatory effects of mCRP on ARPE-19 cells

**Figure 3:**
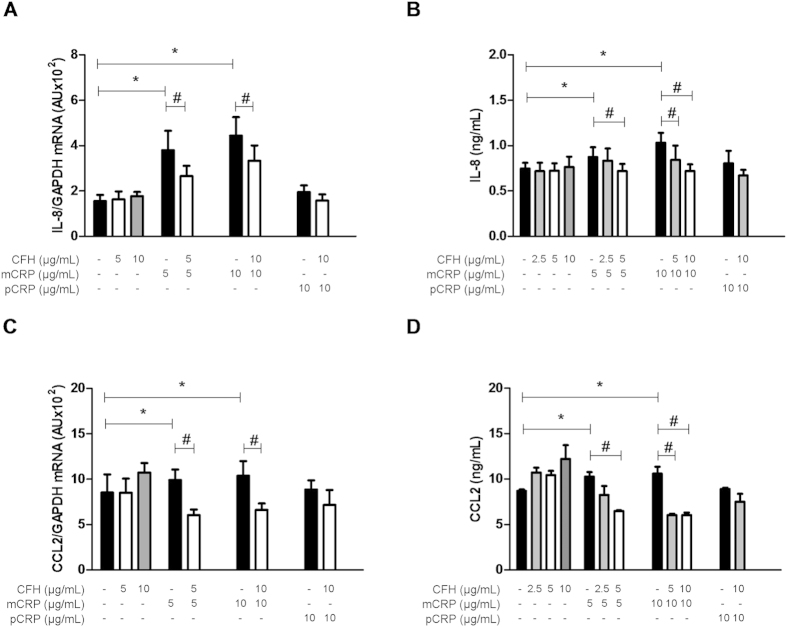
FH counteracts mCRP-induced upregulation of IL-8 and CCL2 in ARPE-19 cells. Cells were stimulated with FH before adding CRP isoforms for 24 h. mRNA expression levels of *IL8* (**A**) and *CCL2* (**C**) were determined using real-time PCR (**A, C**), while concentrations of secreted proteins (**B, D**) were measured by ELISA (n = 5). Statistical analysis was performed by ANOVA (*P < 0.05 vs. control, ^#^*P* < 0.05 vs. mCRP plus FH).

Because FH has been reported to bind CRP, we next evaluated whether FH could prevent the proinflammatory effect of mCRP. For this purpose, ARPE-19 cells were exposed to purified commercial FH 30 min before CRP treatment. Indeed, addition of the complement regulator reduced mCRP-induced upregulation of *IL8* gene expression, although it was not able to completely prevent an increase in *IL8* mRNA levels at the concentrations tested (Fig. 3A). In striking contrast, neither FH alone nor FH added in the presence of pCRP had any effect on IL-8 secretion by ARPE-19 cells. Further, addition of FH prevented mCRP-induced IL-8 secretion in a dose-dependent manner (Fig. 3B). In a similar manner, pre-incubation with FH dose-dependently prevented an increase in *CCL2* mRNA expression upon mCRP stimulation (Fig. 3C) as well as mCRP-induced CCL2 secretion (Fig. 3D). As observed for IL-8, neither FH alone nor FH in the presence of pCRP had any impact on CCL2 secretion.

**Figure 4:**
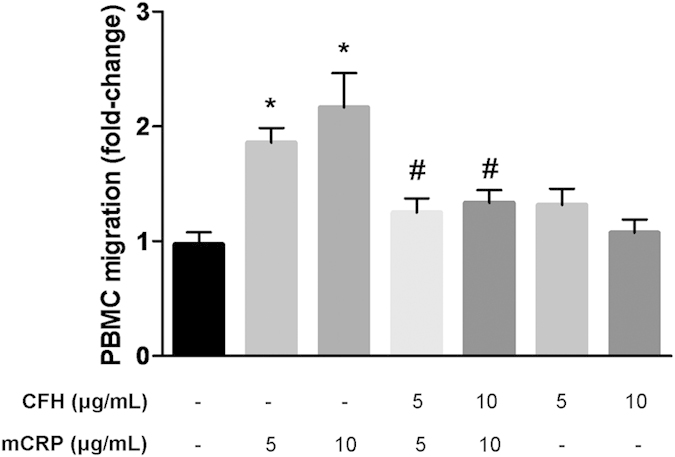
FH counteracts the proinflammatory effect of CRP on ARPE-19 cells. Chemotaxis assay was performed with Transwell chambers. PBMCs from healthy donors were seeded into the upper wells, while the lower chambers contained conditioned medium from ARPE-19 cells stimulated with the indicated concentrations of FH and/or CRP isoforms, and diluted 1:1 with RPMI. Results are expressed as fold-change vs. control ± SEM after 3 h incubation (n = 4). Statistical analysis was performed by ANOVA (*P < 0.05 vs. control, ^#^*P* < 0.05 vs. mCRP).

To further elucidate whether FH could also reverse the enhanced PBMC migration induced by conditioned medium from mCRP-treated ARPE-19 cells, chemotaxis experiments were performed essentially as described above. Medium from ARPE-19 cells treated with FH plus mCRP (5 and 10 μg/mL) induced PBMC migration to a similar extent to that from untreated ARPE-19 cells, and significantly lower than conditioned medium from cells treated with mCRP alone (*P* < 0.05, Fig. 4).

### FH binds to monomeric, but not to pentameric CRP

**Figure 5:**
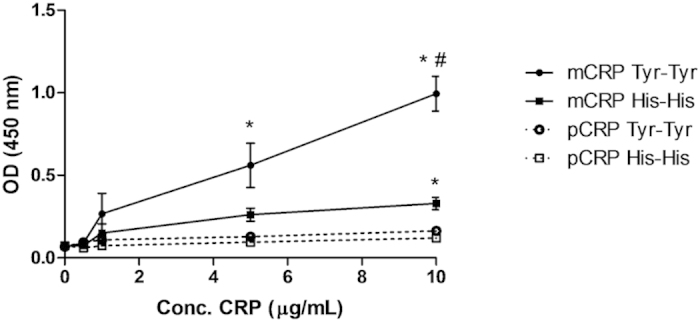
Monomeric CRP, but not pCRP, binds tightly to FH. Various amounts of purified mCRP and pCRP were immobilized on a microtiter plate and serum (1;1,000 diluted) from AMD patients homozygous for the *CFH*_*H402*_ or *CFH*_*Y402*_ variants was added. Bound FH was detected with a polyclonal anti-human FH antibody. Notice that mCRP (solid lines), but not pCRP (dashed lines), bound both FH variants in a dose-dependent manner. Further, mCRP bound tighter to the non-risk FH variant (filled circles, solid line) compared with the risk variant (filled squares, solid line) (n = 3). *P < 0.05 vs. pCRP, ^#^*P* < 0.05 vs. mCRP-FH_Y402_.

We next evaluated the impact of variations at position 402 of FH on the interaction with CRP isoforms. For this purpose, binding of immobilized mCRP or pCRP to FH variants was assessed by ELISA using sera from genotyped AMD patients. FH bound dose-dependently to immobilized, monomeric CRP but not to the pentameric form (Fig. 5). Of particular note, the non-risk associated variant FH_Y402_ bound significantly tighter to mCRP than the risk variant FH_H402_ (*P* < 0.05).

### “Non-risk” but not “at risk” FH prevents the proinflammatory effects of mCRP on ARPE-19 cells

**Figure 6:**
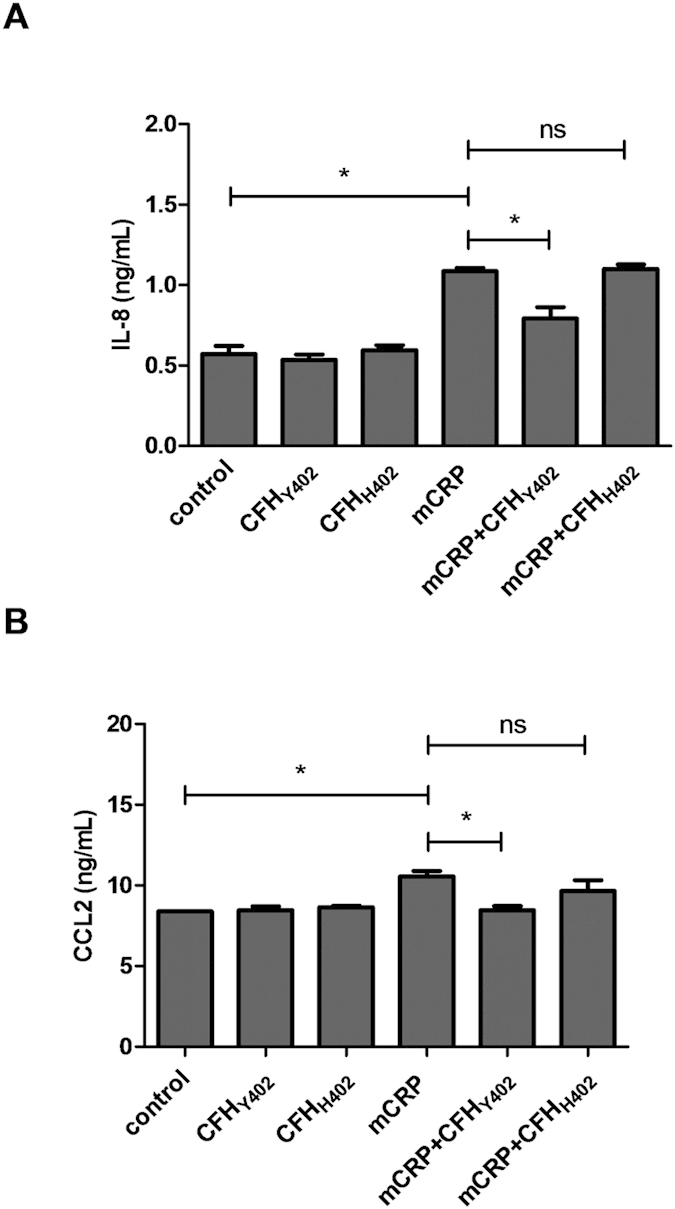
FH_Y402_ purified from sera of genotyped AMD patients counteracts mCRP-induced IL-8 and CCL2 upregulation in ARPE-19 cells more efficiently than the FH_H402_ risk variant. Cells were stimulated with FH purified from plasma of genotyped AMD patients and mCRP isoforms for 24 h. Concentrations of secreted IL-8 (**A**) and CCL2 (**B**) were measured by ELISA (n = 3). Statistical analysis was performed by ANOVA (*P < 0.05).

To test whether the impaired FH-mCRP interaction in individuals carrying the FH_H402_ risk variant could affect the ability of FH to prevent the mCRP-induced pro-inflammatory phenotype, ARPE-19 cells were treated with FH purified from sera of genotyped AMD patients prior to incubation with mCRP. (Because two different commercial antibodies against human FH showed strong cross-reactivity with complement factor C3, we routinely verified authenticity of purified FH by mass spectrometry analysis of tryptic digests; see Supplementary Figure S6 for this quality control). Interestingly, the non-risk associated variant of FH showed a significantly higher ability to prevent mCRP-induced IL-8 and CCL2 up-regulation than the FH_H402_ isoform (Fig. 6).

**Figure 7:**
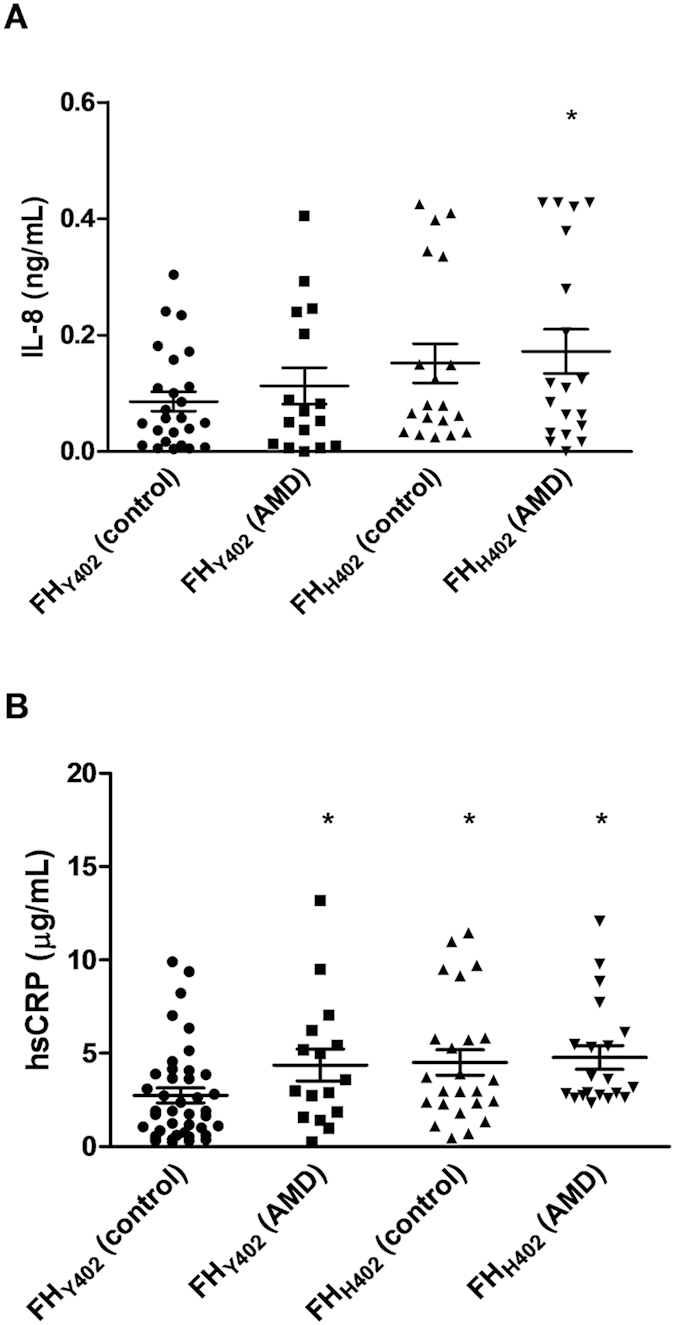
The circulating levels of IL-8 and CRP in AMD patients homozygous for the H402 variant of FH are higher than those of healthy individuals. Serum from healthy subjects homozygous for the non-risk (n = 30) and risk *CFH* alleles (n = 25), as well as from AMD patients homozygous for both alleles (n = 20 in both cases) were collected. Serum levels of IL-8 (**A**) and hsCRP (**B**) were determined by ELISA. Statistical analysis was performed by the Kruskal-Wallis test (*P < 0.05 vs. control).

Finally, to test whether the *CFH*_H402_ risk variant could affect the systemic levels of IL-8, serum concentrations of IL-8 and CRP were determined in genotyped AMD patients and sex- and age-matched controls. AMD patients carrying the risk variant had significantly higher levels of both IL-8 (*P* = 0.03) and hsCRP (*P* = 0.002) than healthy subjects carrying the non-risk variant (Fig. 7). Moreover, the levels of CRP and IL-8 in serum were positively correlated in all subjects (Spearman’s rho r = 0.317, *P* < 0.01) and in particular in the subgroup of AMD patients homozygous for the risk *CFH* variant (r = 0.545, *P* < 0.01). Finally, it is noteworthy that healthy subjects carrying the non-risk variant had significantly lower levels of hsCRP than the other three groups.

## Discussion

Inflammation and immune-mediated processes are crucial for the pathogenesis of AMD and this study strengthens observations that plasma levels of CRP are associated with an increased risk for AMD^10^,^12^,^42^. In the current work, we provide mechanistic insight on how CRP contributes to the development of AMD. First, we show that monomeric CRP but not the pentameric form upregulates the proinflammatory cytokines, IL-8 and CCL2, in retinal pigment epithelial cells. Further, we demonstrate that the regulator of complement responses, FH, binds mCRP to attenuate its proinflammatory activity. Finally, and corroborating cell culture findings, FH from AMD patients carrying the H402 risk variant also displayed impaired binding to mCRP, potentially leaving the proinflammatory effects of mCRP unchecked.

IL-8 and CCL2 are critically involved in choroidal neovascularization (CNV) in AMD^43^,^44^,^45^. They regulate migration and recruitment of leukocytes and regulate the integrity of the blood-retinal barrier, particularly during evolution of CNV and inciting irreversible tissue damage. In the present work we have shown that the pentameric form of CRP does not confer a proinflammatory phenotype to ARPE-19 cells, while mCRP upregulated *IL8* and *CCL2* gene expression and protein secretion in a dose-dependent manner. Notably, this effect was significant at concentrations above 2 μg/mL, which correspond to CRP plasma concentrations predicting risk of AMD in different studies^10^,^12^,^42^. (CRP levels higher than 10 μg/mL are usually attributed to other causes such as acute infection or inflammation). In line with previous studies in endothelial cells^27^, mCRP-induced cytokine production was mediated by p38 MAPK and JNK, since their inhibition partially prevented mCRP effects. Furthermore, the increased secretion of IL-8 and CCL2 enhanced PBMC migration (Fig. 2). Our observations agree with previous studies showing mCRP-induced IL-8 and CCL2 upregulation in endothelial cells and neutrophils^27^,^46^,^47^. By contrast, our results are at odds with a previous report of increased IL-8 expression on ARPE-19 cells upon pCRP stimulation^48^. In this regard, we notice that Wang and colleagues used higher, supraphysiological concentrations of pCRP (up to 100 μg/mL), well above those associated with AMD risk.

In our hands, FH bound the monomeric CRP form rather than pCRP, as previously shown^37^,^38^,^49^. Further, we demonstrated that FH binding to mCRP counteracted its proinflammatory effects (Fig. 3). Notably, conditioned medium from cells treated with both FH and mCRP induced similar PBMC migration as that collected from untreated cells, and significantly lower than medium from mCRP-treated cells (Fig. 4). Of particular relevance when considering the etiology of AMD, we observed that the non-risk variant FH_Y402_ bound more tightly to mCRP than the FH_H402_ risk variant (Fig. 5). Several previous studies have reported similar observations, but with the pentameric CRP form instead^39^,^41^,^50^,^51^. In this regard, it should be noted that immobilized pCRP in the absence of Ca^2+^ ions destabilizes the native fold and pentameric assembly, leading to dissociation^38^. Thus, it seems plausible that previous investigations on pCRP-FH interactions were actually reporting on mCRP-FH binding. In line with these findings, CRP levels in the RPE–choroid layer of individuals who are homozygous for the risk variant are higher than those of individuals homozygous for the non-risk associated form^40^, which could explain the chronic inflammation observed in AMD patients.

To explore the possible binding mode(s) of complement factor H to mCRP, we performed docking experiments taking advantage of previously reported crystal structures of pCRP and FH fragments that include module SCR7. Six out of the ten top docking solutions identified by ZDOCK correspond to arrangements in which the aromatic side chains of Tyr^402^ and the nearby Tyr^390^/Tyr^393^ dock into a groove lined by CRP residues Trp^162^, Phe^164^ and Phe^180^; a histidine side chain at position 402 could not fit this aromatic groove as good as the wild-type tyrosine. A representative solution is presented in Supplementary Figure 1B; it is particularly rewarding that additional interactions of surrounding residues would contribute to complex formation. Most notably, the carboxylate of Glu^101^ (CRP) could engage in simultaneous salt bridge formation with FH lysine residues at positions 388 and 405, while the Arg^188^ and Glu^395^ side chains might form additional salt bridges. Although the W162/F164/F180 crevice on the surface of the CRP molecule is fully accessible in the pentamer, these additional interactions are not possible in the pentameric form, as Glu^101^ faces the intermonomer interface to form a salt bridge with Lys^201^ from a neighboring monomer. This feature might explain why FH preferentially engages mCRP but not pCRP in solution.

The impaired FH-mCRP interaction in individuals carrying the *CFH*_*H402*_ risk variant affected the ability of FH to prevent mCRP-mediated proinflammatory responses, as demonstrated using FH purified from sera of genotyped AMD patients. Remarkably, the non-risk variant of FH showed a significantly higher ability to counteract mCRP-induced IL-8 upregulation than the His402 form. Interestingly, in line with these findings, AMD patients carrying this risk variant of *CFH* had significantly higher levels of IL-8 and serum CRP than healthy subjects carrying the non-risk allele (Fig. 7). Further, the levels of these proteins were positively correlated in AMD patients homozygous for the risk *CFH* variant. Our results do conflict with a previous study showing that CRP levels and the *CFH*_*H402*_ polymorphism were independent risk factors for AMD^52^. We observed differences in hsCRP levels between subjects carrying the different *CFH* variants albeit in a smaller population. The corollary is that our results explain the previously reported higher risk of AMD within genetically susceptible individuals when CRP concentrations are high^49^. Indeed, higher levels of circulating CRP could derive in higher mCRP concentrations in microenvironments that favor dissociation (i.e. inflammatory or apoptotic conditions), which in the case of patients carrying the *CFH*_*H402*_ risk variant would further cause unchecked inflammation.

Recently, a specific pathophysiological role for mCRP in chronic inflammatory disorders such as cardiovascular disease emerged^22^,^28^,^29^. In the context of AMD the presence of both CRP isoforms in ocular tissues has also been recently reported^21^. Notably, mCRP has been identified in AMD tissues along Bruch’s membrane and also in drusen, supporting the relevance of mCRP for the disease pathogenesis. Extrahepatic synthesis of CRP in different cell types has been reported^53^,^54^. However, we were unable to detect *CRP* gene expression in ARPE-19 cells although we have not confirmed this in primary cultures. Alternatively, mCRP can be generated locally from dissociation of pCRP on the surface of necrotic or activated cells^21^,^22^,^28^. This process appears to depend on lysophosphatidylcholine exposure after phospholipase A2 activation as elegantly demonstrated by Thiele and colleagues in an *in vivo* animal model of acute cardiovascular inflammation^24^.

**Figure 8:**
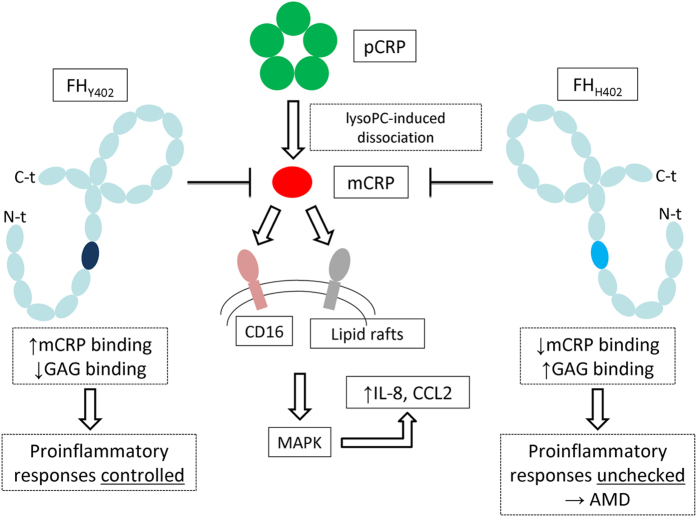
A unified mechanism of mCRP-induced proinflammatory responses and the role of the *CFH* p.Tyr402His polymorphism. Generation of mCRP can occur spontaneously, but is accelerated *in vivo* under pathological conditions by bioactive lipids such as lysophosphatidylcholine (lysoPC) exposed on the surface of activated or damaged cells. mCRP is recognized by CD16 and/or other receptor(s) on the cell surface, leading to activation of MAPK pathways, and ultimately to enhanced expression of proinflammatory cytokines. Binding of FH to mCRP attenuates this inflammatory response, but the FH_H402_ variant is less effective in this regard, both because it binds tighter to glycosaminoglycans (GAG), but in particular due to its markedly lower affinity for mCRP. (Notice that FH domain SCR7, which contains residue 402 is colored differently in the two variants). The unchecked inflammatory response leads eventually to neovascularization, progression of AMD, and finally vision loss.

A previous work concluded that the association between the p.Tyr402His *CFH* polymorphism and AMD was due to a tighter association of the His402 variant with sulfated glycosaminoglycans (GAGs)^55^. In contrast, we demonstrate here a weaker binding of FH_H402_ to mCRP as a major factor underlying retinal degeneration. The two explanations are only apparently contradictory, however, and it is conceivable that both effects contribute to the etiology of AMD. In particular, it is evident that GAG-bound FH would not be able to complex mCRP generated *in situ*, as the two FH ligands share partially overlapping binding epitopes centered on residue 402. A mechanism that unifies currently available information on the role of mCRP-FH interactions for AMD progression is schematically presented in Fig. 8.

The main limitation of our study is the use of a spontaneously arising RPE cell line, ARPE-19. Although ARPE-19 cells behave in many ways like primary RPE cultures this may not be always the case, and caution should be exercised when extrapolating our current results to an *in vivo* setting. Nevertheless, it must be stressed that ARPE-19 cells are commonly used for studying oxidative stress and cell signaling in AMD, as they show features of aged RPE. Therefore, the results reported herein appear valid as a first attempt to evaluate the role of mCRP on RPE cells *in vitro*.

In summary, our work shows that CRP is not only a risk marker but also is likely to contribute to the progression of AMD. Furthermore, our results highlight the importance of CRP quaternary structure on its function, as the monomer, but not the pentameric form, induced a proinflammatory response in a human RPE cell line. We also demonstrated that the proinflammatory effect of mCRP is not regulated by FH from subjects that carry the risk variant His402 of the complement regulator, due to an impaired interaction with mCRP. The ability of FH to bind mCRP and counteract the proinflammatory activity should be explored further by elucidating the structural basis of FH-mCRP complex formation.

Our current findings and those of other authors reinforce the importance of mCRP in inflammatory disorders and point to the CRP dissociation process and to mCRP-FH interactions as novel therapeutic targets. Along these lines, inhibition of pCRP dissociation with 1, 6-bis-PC, a compound that stabilizes CRP in a decameric form, abolished the proinflammatory effects of mCRP *in vivo*^19^. In a similar manner, therapeutic approaches aimed to enhance FH-mCRP binding could be developed to block mCRP proinflammatory activities, thus preventing the progression of AMD. Future research is warranted to confirm the contribution of mCRP to the disease etiology and progression and eventually to test the therapeutic potential of compounds that either prevent CRP dissociation or stimulate FH binding to mCRP in animal models.

## Materials and Methods

### Materials

High purity human pCRP and FH were purchased from Calbiochem. Kits for DNA or RNA isolation were from QIAGEN, and M-MuLV reverse transcriptase from Invitrogen. Antibiotics, amino acids and culture media were from PAA, and plates from Nunc. Transwell inserts were obtained from Corning. Chromatography resins were bought from GE Healthcare Life Sciences. Unless otherwise stated, all other reagents, of the highest purity commercially available, were purchased from Sigma-Aldrich or GE Healthcare.

### Patients

Patients with wet AMD (20 females and 20 males, mean age = 74) and controls (29 females and 26 males, mean age = 71) were of Caucasian origin and were recruited at the Department of Ophthalmology of the Hospital Clínic of Barcelona. All patients and controls provided informed consent, and the research followed the tenets of the Declaration of Helsinki. The Hospital Clínic of Barcelona Institutional Review Board (IRB) approved this study according to local and national IRB guidelines. Blood was collected from patients and controls, and serum was prepared and stored at -70 °C until further analysis. Genomic DNA was extracted from peripheral blood using QIAmp DNA Blood Mini Kit. Exon 9 of the *CFH* gene (NM_000186), was amplified by polymerase chain reaction (PCR). PCR amplicons were purified with Illustra ExoStar 1-Step kit (GE Healthcare), bidirectionally sequenced using ABI BigDye^®^ Terminator v3.1 Cycle Sequencing Kit (Applied Biosystems) and run on an automated ABI 3730XL DNA analyzer.

### Generation and handling of mono- and pentameric CRP forms

Native human pCRP was stored in 10 mM Tris, 140 mM NaCl buffer (pH 8.0) containing 2 mM CaCl_2_ to prevent spontaneous dissociation of the native pentamer. mCRP was prepared by urea chelation from purified human CRP as previously described^29^. Briefly, pCRP at 1 mg/mL was chelated with 10 mM ethylene diaminetetraacetic (EDTA) and incubated in 8.0 M urea for 6 h at 37 °C. Urea was then removed via dialysis against low ionic strength TBS (10 mM Tris-HCl, 50 mM NaCl, pH 7.3). mCRP concentration was determined by the BCA protein assay. The filtered solution was stored at 4 °C. Because commercial CRP preparations can be contaminated with sodium azide or lipopolysaccharide (LPS)^28^, pCRP was also dialyzed into TBS before cell experiments.

### FH purification from serum of genotyped individuals

For purification of FH_Y402_ and FH_H402_ variants we followed the recently reported procedure, of Brandstätter and co-workers^56^, with slight modifications. Briefly, serum samples from patients homozygous for each of the FH variants (1 mL) were centrifuged at 15,000 rpm for 2 min, supernatants were filtered (0.45 μm), and then Triton X-100 and tributyl phosphate were added to reach final concentrations of 1 and 0.325%, respectively. These solutions were incubated with stirring for 1 h at 4 °C, and then incubated for 45 min at 4 °C with 1 mL SP-Sepharose Fast Flow equilibrated in 20 mM sodium citrate, pH 6.0. The matrix was washed with citrate buffer containing 100 mM NaCl, and FH finally eluted with 200 mM NaCl. FH fractions were pooled, dialyzed against 25 mM Tris-HCl, pH 8.6 using a Centricon 50 K (Millipore), and incubated for 45 min at 4 °C with 1 mL Q-Sepharose Fast Flow equilibrated with the same buffer. FH was eluted with Tris-HCl buffer containing 250 mM NaCl. For cell experiments, fractions containing FH were dialyzed against 50 mM sodium phosphate buffer, pH 7.5, and concentrated on a Centricon 50 K. The identity and purity of FH variants was verified by SDS-PAGE and Western blot using an anti-human FH (AbD Serotec) and by mass-spectrometry analysis of tryptic digests.

### ARPE-19 cell culture

The spontaneously arising human RPE cell line, ARPE-19, was obtained from the American Type Culture Collection (ATCC). ARPE-19 cells were cultured in a 50:50 mixture of Dulbecco’s modified Eagle medium (DMEM) and Ham’s F12 supplemented with 10% fetal bovine serum (FBS), 2 mM L-glutamine, 100 U/mL penicillin, 0.1 mg/mL streptomycin, and 1 mM sodium pyruvate in a humidified incubator at 37 °C in 5% CO_2_. Cells were passed every 4 to 5 days by trypsinization.

For cytokine stimulation cell suspensions of 1 × 10^5^ cells/mL were seeded onto 24-well tissue culture plates and incubated overnight. Cells were then washed gently with phosphate-buffered saline (PBS) to remove cell debris, and starving fresh culture medium with 0.6% FBS was introduced for 18 h. Cells were then stimulated or not with human CRP isoforms (2–50 μg/mL) for 4–48 h. The responses to mCRP were also studied in the presence of mitogen activated protein kinase (MAPK) inhibitors. SB302580 (5 μM), U0126 (1 μM), and SP600125 (20 μM) were used to inhibit p38 MAPK, MAPK kinase 1 and 2 (MEK1/2), and c-Jun N-terminal kinase (JNK), respectively. Additionally, experiments were also performed in the presence of antibodies capable of blocking Fcγ receptors (2.5 μg/mL of function-blocking anti-CD16 and/or anti-CD32 antibodies; Pharmingen). When indicated cells were treated 30 min before the addition of CRP isoforms with commercial human FH or with FH purified from sera of AMD-genotyped patients.

To explore CRP induction in ARPE-19 cells, cell suspensions of 1 × 10^5^ cells/ml were seeded onto 24-well tissue culture plates and incubated overnight to allow the formation of a monolayer reaching 80% confluence. Cells were then stimulated with different combinations of proinflammatory cytokines: IL-17 (100 ng/mL), IL-1β (50 ng/mL), IL-6 (50 ng/mL), and/or TNF-α (50 ng/mL) for 24–48 h.

### Quantitative real-time PCR

One μg of RNA from cell lysates extracted using RNeasy isolation kit was reverse- transcribed with M-MuLV reverse transcriptase for 1 hour at 42 °C in a total volume of 25 μL. *IL8*, *CCL2*, and *CRP* mRNA levels were analyzed by real-time PCR. Assays on demand (Applied Biosystems) were used for IL-8 (Hs00174103m1), CCL2 (Hs00234140m1), and CRP (Hs04183452g1). Human GAPDH was used as endogenous control. Quantitative real-time PCR was performed on an ABI PRISM 7000 Detection System. The comparative CT method was used to determine the relative fold changes in gene expression.

### Enzyme-linked immunosorbent assay (ELISA)

Supernatants from CRP-stimulated cells were collected and centrifuged (1,000 × g for 15 minutes) to remove particulates, and stored at −70 °C until further analysis. Protein concentrations of IL-8 and CCL2 were measured using human ELISA development kits (Ready Set Go eBiosciences). Each stimulation experiment was repeated five times. CRP levels in the cell supernatants were quantified using a commercial high-sensitivity ELISA kit specific for human CRP (hsCRP, IBL International GmbH; minimum detectable concentration 0.4 ng/mL) according to the manufacturer’s instructions. These experiments were performed in triplicate.

### Isolation of human peripheral blood mononuclear cells (PBMC)

Human PBMC were isolated from whole blood of healthy donors on a density gradient (Ficoll-Paque PLUS). PBMC were washed twice in RPMI 1640 medium containing 2% FBS and resuspended at 2 × 10^6^ cells/mL in PBMC starving medium (RPMI 1640 containing 20 mM Hepes, 1% FBS, 2 mM L-glutamine, 100 U/mL penicillin, and 0.1 mg/mL streptomycin).

### Chemotaxis assay

Chemotaxis assays were performed according to published methods. Conditioned media of ARPE-19 cells treated with different CRP isoforms diluted 1:2 with PBMC starving medium were used in the assay. Alternatively, PBMC starving medium supplemented with either 10 μg/mL of pCRP or mCRP was also used for chemotaxis experiments. Transwell inserts (6.5 mm in diameter, 5-μm pore) were placed in 24-well plates. One-hundred μL of a human PBMC suspension containing 2 × 10^5^ cells and 100 μL of diluted conditioned medium or PBMC starving medium supplemented with CRP isoforms were added to the top and bottom compartments of each insert, respectively. PBMC were allowed to migrate for 3 h at 37 °C in a standard tissue culture incubator. After this period, the Transwell inserts were removed, cells were collected, and cells migrated to the bottom compartment were counted in a Neubauer chamber.

### Binding of FH variants to CRP isoforms

Maxisorp microtiter plates were coated with CRP isoforms (0.5–10 μg/mL) overnight at 4 °C. After blocking, serum (1:1,000) from patients homozygous for the *CFH*_*H402*_ or *CFH*_*Y402*_ variants was added. The plates were incubated for 1 h at 37 °C and washed twice. Biotinylated polyclonal anti-human FH (AssayPro human FH ELISA kit) was added, followed by the addition of streptavidin-peroxidase conjugate (AssayPro human FH ELISA kit). Chromogenic substrate was finally added, and absorbance was measured at 450 nm as indicated by the manufacturer.

### Statistical analysis

Results were expressed as mean ± SEM. After testing for normal distribution and equality of variances with Levene *F* test, Student *t*, ANOVA or Kruskal Wallis tests as appropriate were used to determine statistical significance between treatments. A value of *P* < 0.05 was considered significant. All calculations were performed using SPSS Version 18.0 (SPSS, IBM).

## Additional Information

**How to cite this article**: Molins, B. *et al.* Complement factor H binding of monomeric C-reactive protein downregulates proinflammatory activity and is impaired with at risk polymorphic CFH variants. *Sci. Rep.* **6**, 22889; doi: 10.1038/srep22889 (2016).

## Supplementary Material

Supplementary Information
