# Supplementary material for: Complement factor H binding of monomeric C-reactive protein downregulates proinflammatory activity and is impaired with at risk polymorphic CFH variants
Source: Sci Rep. 2016 Mar 10;6:22889. doi: 10.1038/srep22889 (PMC4785391; doi:10.1038/srep22889)
Supplement: Supplementary Information [file srep22889-s1.pdf]

Complement factor H binding of monomeric C-reactive protein downregulates proinflammatory activity and is impaired with at risk polymorphic FH variants

Blanca Molins[1,*], Pablo Fuentes-Prior[2,3], Alfredo Adán[1], Rosa Antón[2], Juan I. Arostegui[4], Jordi Yagüe[4], Andrew D. Dick[5,6]

Supplementary Information

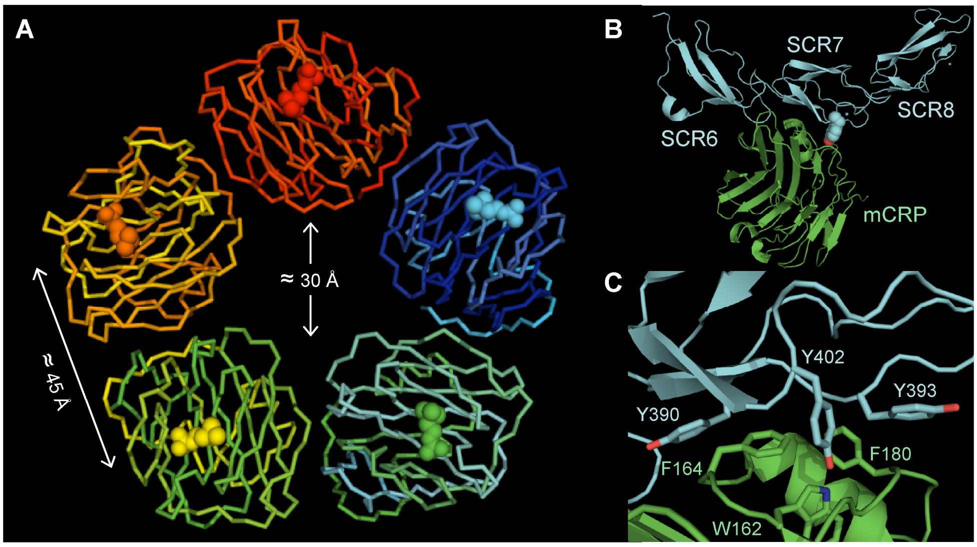

Molins, Supplementary Figure S1

Supplementary Figure S1. Structure of pentameric CRP and putative binding mode of monomeric CRP to complement factor H. (**A**) Crystal structure of pCRP. For simplicity, the five independent monomers are represented by their Cα traces (PDB code 1B09; ref. Thompson *et al.*, Structure 1999). Notice the channel left between the subunits. The phosphocholine molecules bound to each of the Ca^{2+}-dependent binding sites are shown as van der Waals spheres with all their non-hydrogen atoms. (**B**) Representative docking solution of FH to monomeric CRP. The model of the CRP monomer used for docking has been derived from that of the pentamer (panel A) with minor modifications. (Basically, rotation of acidic aspartate / glutamate side chains that coordinate Ca^{2+} ions in pCRP to attenuate electrostatic repulsion). Docking of FH domain SRC7 was performed with ZDOCK. No residues were excluded from binding and only FH residue Tyr^{402} was explicitly forced to contact the CRP molecule. Surprisingly, six out of the ten top docking solutions revealed that the side chain of Tyr^{402}, together with the pair of nearby tyrosines at positions 390 and 392, occupy an aromatic cage lined by the CRP side chains of Trp^{162}, Phe^{164} and Phe^{180}. A close-up of the putative complex around the Tyr^{402} side chain is shown in panel (**C**).

A

B

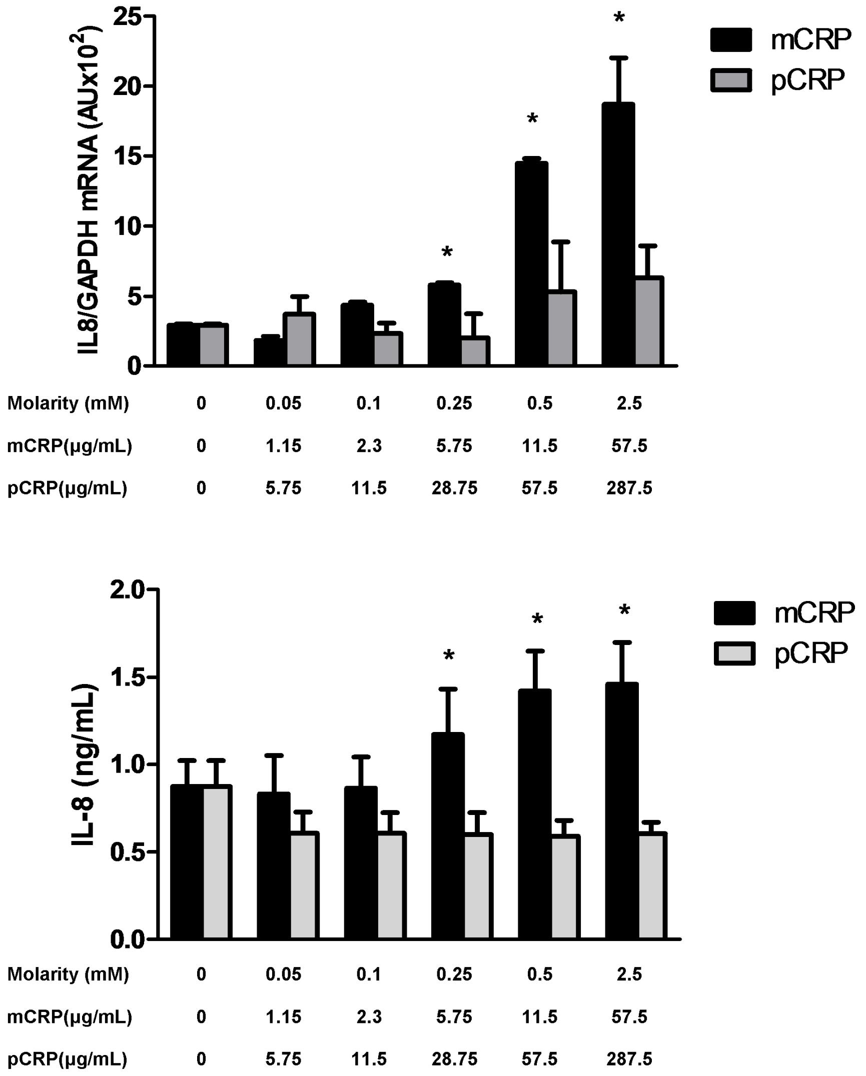

Molins, Supplementary Figure S2

Supplementary Figure S2. Monomeric, but not pentameric CRP stimulate IL-8 expression. ARPE-19 cells were stimulated with the indicated molar concentrations of CRP isoforms for 24 h. Gene expression levels (A) and secreted concentrations of IL-8 (B) were determined by real-time PCR and ELISA, respectively (n = 3). Statistical analysis was performed by ANOVA (**P* < 0.05 vs. control and pCRP).

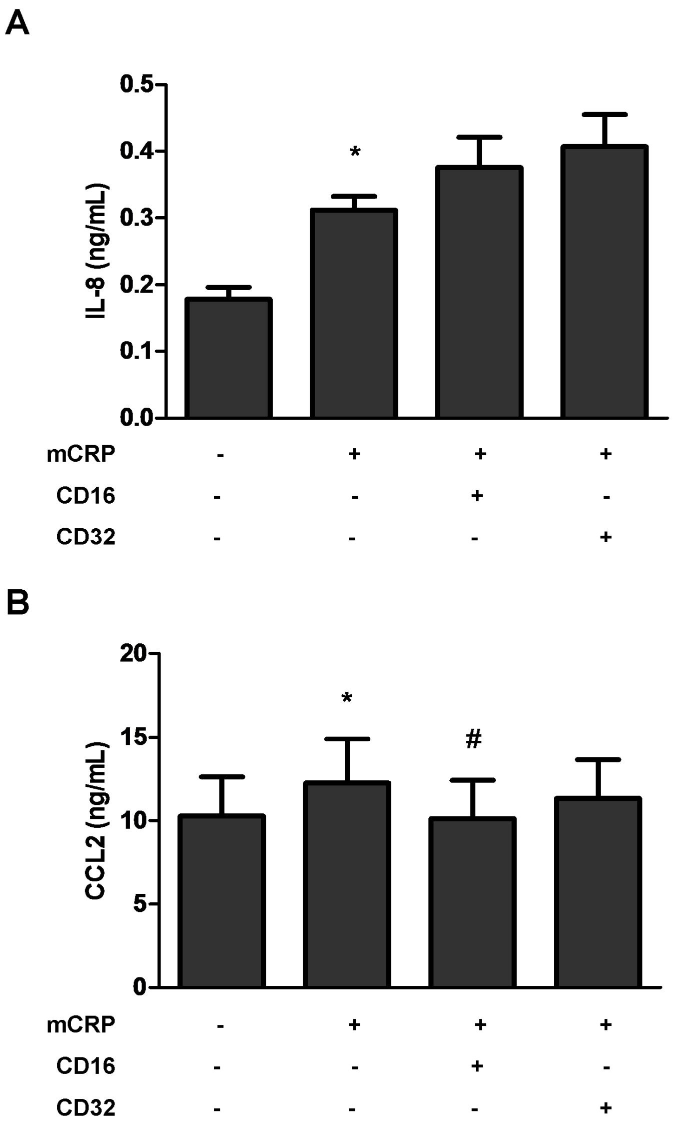

Molins, Supplementary Figure S3

Supplementary Figure S3. Fcγ receptor CD16, but not CD32, is involved in mCRP-induced stimulation of IL-8 and CCL2 secretion. ARPE-19 cells were treated with blocking function antibodies against CD16 (2.5 µg/mL) or CD32 (2.5 µg/mL) before mCRP treatment for 24 h, and secreted levels of IL-8 (**A**) and CCL2 (**B**) were determined by ELISA (n = 4). Statistical analysis was performed by ANOVA (*$P < 0.05$ vs. control, #$P < 0.05$ vs. mCRP).

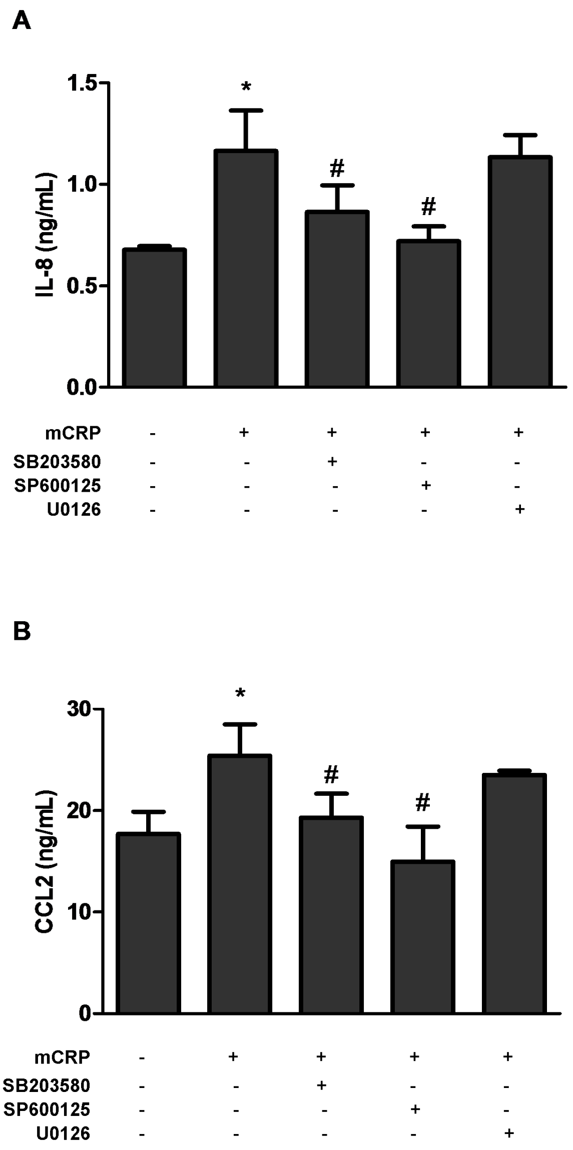

Molins, Supplementary Figure S4

Supplementary Figure S4. Inhibition of p38 MAPK or JNK but not of MEK ½ reduces mCRP-induced secretion of IL-8 and CCL2. ARPE-19 cells were treated with SB302580 (5 µM), U0126 (1 µM), or SP600125 (20 µM) prior to mCRP treatment, and secreted levels of IL-8 (**A**) and CCL2 (**B**) was determined by ELISA (n = 4). Statistical analysis was performed by ANOVA (**P* < 0.05 vs. control, #*P* < 0.05 vs. mCRP).

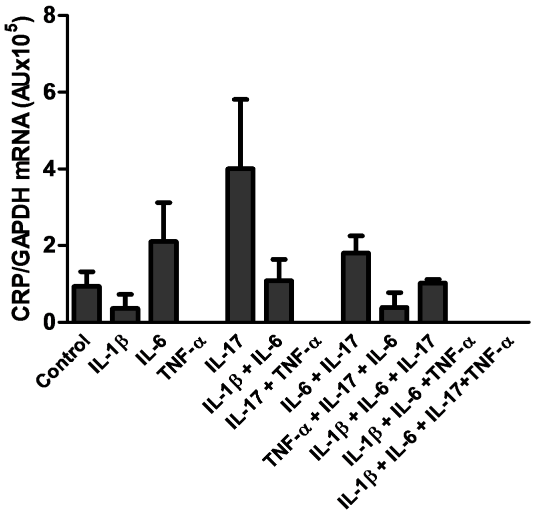

Molins, Supplementary Figure S5

Supplementary Figure S5. *CRP* is not expressed in ARPE-19 cells. Cells were treated for 24 h with different combinations of proinflammatory cytokines, and *CRP* mRNA levels were determined by real time RT-PCR (n = 3).

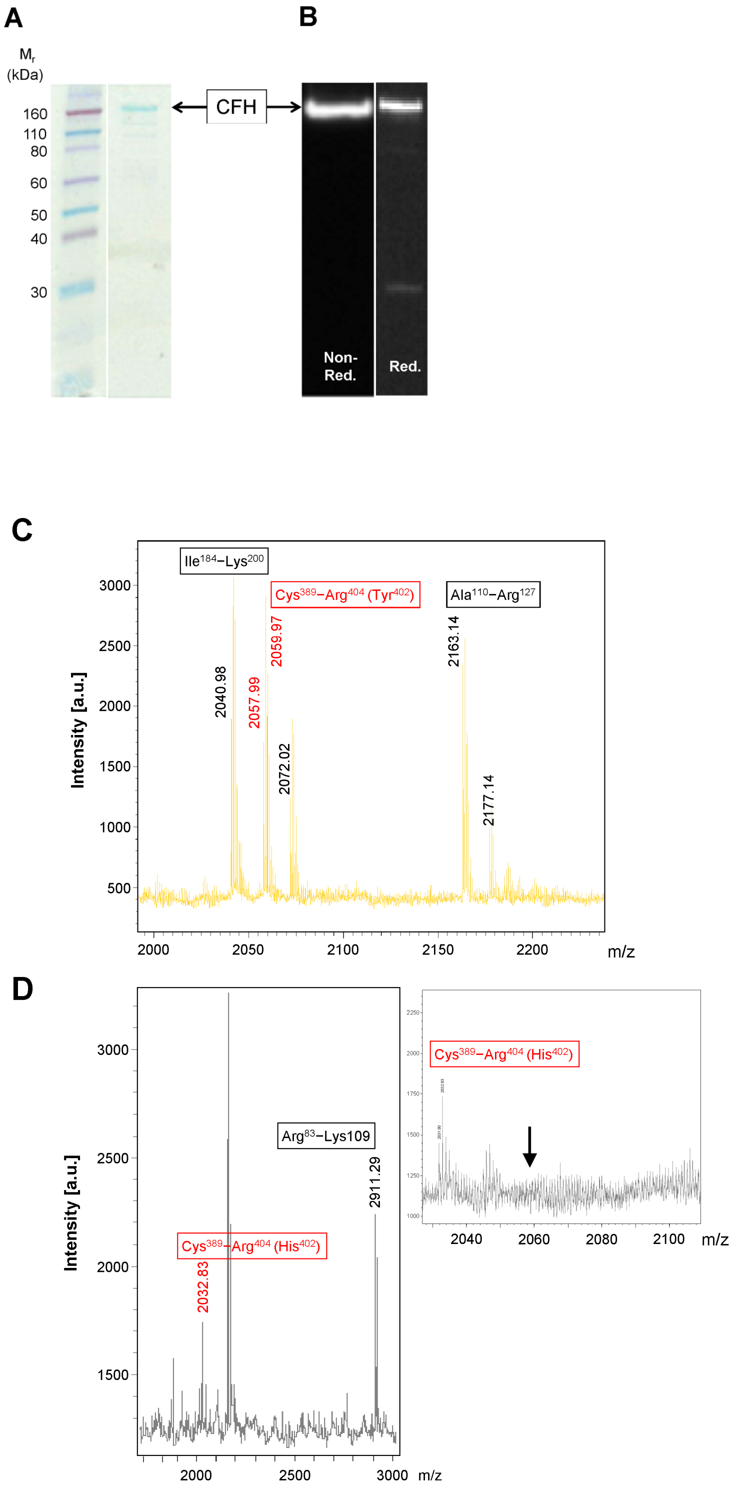

Molins, Supplementary Figure S6

Supplementary Figure S5. FH$_{Y402}$ and FH$_{H402}$ variants can be purified from plasma of genotyped patients. FH variants were purified following the protocol reported by Brandstätter

and co-workers, with modifications. Purity and identity of the purified protein were verified by SDS-PAGE (**A**), Western blot (**B**) and mass spectrometry analysis of tryptic digests of purified FH_{H402} **(C)** and FH_{Y402} **(D).** Notice in particular the identification of a peak that corresponds to the Cys389-Arg404 fragment of human FH, which therefore includes residue 402. In mass spectra of Y402 FH there is no peak that would correspond to the H402 variant and vice versa, demonstrating lack of cross-contamination of the purified FH variants.
